# Adult judgments of children’s pain and fear during venipuncture: The impact of adult and child sex

**DOI:** 10.1080/24740527.2018.1537672

**Published:** 2018-11-08

**Authors:** Meghan G. Schinkel, Katelynn E. Boerner, Christine T. Chambers, C. Meghan McMurtry

**Affiliations:** a Department of Psychology and Neuroscience, Dalhousie University, Halifax, Nova Scotia, Canada; b Centre for Pediatric Pain Research, IWK Health Centre, Halifax, Nova Scotia, Canada; c Department of Pediatrics, Dalhousie University, Halifax, Nova Scotia, Canada; d Department of Psychology, University of Guelph, Guelph, Ontario, Canada; e Pediatric Chronic Pain Program, McMaster Children’s Hospital, Hamilton, Ontario, Canada; f Department of Paediatrics, Western University, London, Ontario, Canada

**Keywords:** pediatric pain, pain assessment, fear assessment, sex, pain catastrophizing

## Abstract

**Background:**

Low levels of agreement between caregiver and child reports of acute pain are well documented.

**Aims:**

This study builds on prior research through exploring factors that may contribute to low caregiver–child concordance. Specifically, the study examined the influence of adult and child sex on adult judgments of children’s pain and fear during venipuncture and examined whether trait parental pain catastrophizing, empathy, and anxiety predicted judgment accuracy.

**Methods:**

Using a judgment study paradigm, 160 participants (82 women) viewed 20 10-s video clips of children (10 boys, 10 girls) undergoing venipuncture and rated each child’s pain and fear. Adults’ ratings were compared to the children’s own ratings. Adults completed measures of trait parental pain catastrophizing, dispositional empathy, and trait anxiety.

**Results:**

Adults accurately judged boys’ pain and fear significantly more often than that of girls. Further, adults underestimated and overestimated girls’ pain and overestimated girls’ fear significantly more frequently than that of boys. No effects of adult sex or adult by child sex interactions emerged. Parental pain catastrophizing significantly predicted underestimation of girls’ pain, with adults who engaged in more catastrophizing being less likely to underestimate girls’ pain. The variables did not predict adult judgment of child pain for women and men separately and did not predict adult judgment of child fear when examined by adult sex, child sex, or both combined.

**Conclusions:**

Child sex influences adult pain and fear judgments, with girls being more vulnerable to inaccurate assessment than boys. Higher levels of parental pain catastrophizing may buffer against adults’ propensities to underestimate girls’ pain.

## RÉSUMÉ

**Contexte**: Le faible niveau de concordance entre la douleur aiguë rapportée par le prestataire de soins et celle rapportée par l’enfant est bien documentée.

**But**: Cette étude s’appuie sur des études antérieures pour examiner les facteurs qui peuvent contribuer à la faible concordance prestataire de soins-enfant. Plus particulièrement, cette étude portait sur l’influence du sexe de l’adulte et celui de l’enfant sur les jugements que portent les adultes sur la douleur et la peur de l’enfant pendant une ponction veineuse et examine si la catastrophisation de la douleur par le parent, l’empathie et l’anxiété prédisaient l’exactitude de ces jugements.

**Méthodes**: À l’aide d’un paradigme pour l’étude des jugements, 160 participants (82 femmes) ont visionné 20 clips vidéo de 10 secondes présentant des enfants (10 garçons, 10 filles) au moment d’une ponction veineuse, et ont attribué une note à la douleur et à la peur ressentie par l’enfant. Les notes attribuées par les adultes ont été comparées à celels attribuées par les enfants eux-mêmes. Les adultes se sont soumis à la mesure de la catastrophisation de la douleur en tant que trait parental, de leur tendance à l’empathie et de l’anxiété chronique.

**Résultats**: La adultes ont jugé avec exactitude la douleur et la peur ressenties par les garçons significativement plus souvent que celles ressenties par les filles. De plus, les adultes ont sous-estimé et surestimé la douleur des filles, et surestimé la peur des filles significativment plus fréquemment que celle des garçons.. Aucun effet du sexe de l’adulte ni aucune interaction entre le sexe de l’adulte et celui de l’enfant n’a été observé. La catrastrophisation de la douleur par le parent prédisait significativement la sous-estimation de la douleur ressentie par les filles, alors que les adultes qui catastrophisaient davantage étaient moins susceptibles de sous-estimer la douleur des filles. Les variables ne prédisaient pas le jugement de la douleur des enfants par les adultes pour les femmes et les hommes séparément, et ne prédisaient pas le jugement de la peur de l’enfant par les adultes lorsqu’examiné par sexe de l’adulte, sexe de l’enfant ou une combinaison des deux.

**Conclusions**: Le sexe de l’enfant influence le jugement de l’adulte sur la peur et la douleur, les filles étant plus vulnérables aux jugements inexacts que les garçons. Des niveaux plus élevés de catastrophisation de la douleur par le parent pourraient amortir la propension des adultes à sous-estimer la douleur ressentie par les filles.

Although children often rely on adults for pain assessment, research suggests that parents’ ratings of children’s pain are not always in agreement with the child’s own ratings, and parents often underestimate children’s pain.^1–6^ Further, low agreement between parent and child fear ratings following venipuncture has been documented.^7^ A broad approach to assessing child pain, which may include adult caregivers’ perspectives, is advocated when determining treatment.^8^ Therefore, adult pain assessment has implications for children’s care. Given the importance of accurate pain and fear assessment to guide necessary intervention, the reasons why adults are inaccurate need to be explored.^9,10^

Reviews have demonstrated that pain perception and assessment can be impacted by several factors, including the type of caregiver assessing pain (relative versus health care provider) and the type of pain being assessed (acute versus chronic),^10^ as well as an individual’s prior exposure to his or her own and others’ pain, the age of the individual experiencing pain, and the positivity of the relationship between the assessor and individual in pain.^11^ Regarding child pain specifically, adult sex may impact child pain assessment in terms of accuracy and intensity. Moon and colleagues found that fathers generally demonstrated better agreement with their child’s pain ratings during an experimental pain task than mothers.^3^ Child sex also differentially influenced mothers’ and fathers’ ratings of child pain, with fathers participating with a son rating their child’s pain as being more intense than fathers participating with a daughter; there was no effect for mothers.^3^ Further, Cohen and colleagues asked adults to view a video of a child of ambiguous gender undergoing a finger-stick blood test and found that adults rated the child as experiencing more pain when told that the child was a boy versus a girl.^12^

Psychological factors, such as dispositional empathy, pain catastrophizing, and anxiety, have been implicated in adult responses to children’s pain and fear. Fear is considered an “in-the-moment” response, whereas anxiety is an emotional experience generally concerned with the future.^7,13,14^ The empathy for pain model describes how an individual’s personal characteristics may influence how one perceives and responds to another’s pain.^15^ In a vignette study, parents who endorsed greater levels of catastrophizing about their child’s pain and higher dispositional empathy had greater personal (e.g., worry, anxiety) and outward-focused (e.g., compassion) emotional reactions to their child being in pain.^16^ Greater parental pain catastrophizing is related to parents having better agreement with their child’s self-reported pressure pain.^4^ One study found that parental anxiety was positively related to parents’ ratings of their child’s immunization pain, and this was mediated by parent ratings of their child’s procedure-related anxiety (or fear).^17^ Thus, pain catastrophizing, dispositional empathy, and anxiety may be relevant variables in understanding adult caregiver pain and fear judgments.

The first objective of the current study was to examine the impact of adult and child sex on judgments of children’s pain and fear during venipuncture. It was hypothesized that men would be more accurate at judging child pain and fear than women and that an adult by child sex interaction would exist, with men displaying greater accuracy in assessing boys’ pain than girls’ pain.^3^ The second study objective was to examine whether adult catastrophizing about children’s pain, trait anxiety, and empathy predicted judgment accuracy. It was hypothesized that each of the identified adult traits would significantly predict judgment accuracy,^4,16,17^ with greater levels of catastrophizing about children’s pain, trait anxiety, and empathy being related to greater judgment accuracy. To examine these factors in a controlled manner, a standardized judgment study paradigm was used in which adults viewed videos of children undergoing venipuncture that systematically manipulated child sex and pain level. In keeping with definitions of accuracy used in prior caregiver judgment research, a participant rating was considered accurate if he or she circled the same face on the pain or fear scale as the child had circled.^1^ Although this is a conservative definition of judgment accuracy, it has been proposed that a one-face difference on the Faces Pain Scale–Revised (the measure of pain intensity used in the current study) may have clinical significance.^18^ Therefore, over- or underestimation by one face may be clinically meaningful to a child’s pain treatment. Further, strict definitions of agreement have been suggested for judgment studies,^2^ because correlational investigations of caregiver–child concordance in pain^2^ and fear^7^ ratings have been found to overstate agreement relative to more stringent approaches.

## Methods

### Participants

Participants were 82 women and 78 men who were parents of generally healthy 6- to 8-year-old children and participated in a separate study exploring the role of sex in social modeling in pediatric pain (see Boerner and colleagues^19^). It should be noted that these participants were unrelated to the children in the video whose pain and fear they were assessing. Participants were recruited from the local community using advertisements, social media, and the research center’s database of previous participants who had agreed to be contacted about future research. In addition to recruitment materials aimed at parents generally, materials were developed that specifically targeted fathers in order to recruit a large sample of male caregivers. For example, study posters were developed that advertised for fathers and children to participate in the study. Data collection occurred between October 2013 and June 2015. Women had a mean age of 37.77 years (range = 20–48; SD = 5.51), and men had a mean age of 40.75 years (range = 26–53; SD = 5.48); men were significantly older than women, *t*(157) = 3.42, *P* < 0.01. With regard to marital status, 67.1% of women and 85.9% of men indicated that they were married. There was a significant relationship between participant sex and marital status, χ^2^(4) = 10.27, *P* < 0.05, suggesting that more men were married than women. The majority of participants reported their race/ethnicity as white (85.6%). The most frequently indicated household income bracket was $75,000–$100,000 (21.3%; range = <$10,000 [1.9%] to >$150,000 [15.6%]), and the majority of participants were either university graduates (30.0%) or had graduate school/professional training (31.9%). Pearson’s chi-square tests revealed no significant relations between participant sex and race, household income, or education.

Inclusion criteria included the participant being a mother or father of a child aged 6–8 years old (who they live with at least 50% of the time); being able to speak, write, and read English fluently enough to answer written questions and engage in conversation; as well as having no uncorrected vision or hearing impairments. Of 179 participants who were enrolled in the larger study, 15 (8%) were excluded due to not completing all study components (e.g., not completing the pain rating task or all of the questionnaire items), three (2%) were excluded because the family decided to withdraw from the study, and one (0.6%) participant was excluded because the accompanying caregiver was not the child’s mother or father.

### Procedure

The study was approved by the Izaak Walton Killam (IWK) Health Centre Research Ethics Board and involved one visit to the research center. Participants provided informed consent and then completed a series of questionnaires, including measures of general demographics, trait anxiety, empathy, and parental catastrophizing about their own child’s pain. Participants then completed a variety of tasks relating to the larger study, including completing a cold pressor experimental pain induction themselves and watching their child also do the pain task (the results of which are described in a separate manuscript^19^).

Lastly, participants were asked to complete a pain rating video task on a laptop. During this task, participants were shown 20 10-s video clips of children undergoing venipuncture. In order to standardize the clips across participants, each of the 10-s video clips captured the period spanning immediately before to just after the insertion of the needle and included sound. Consistent with other observer pain judgment studies,^1^ participants were randomly assigned to view one of five predetermined order sequences of the 20 video clips (the order of the video clips had been randomized for each of the five sequences) to account for potential order effects. Participants were only allowed to view each video clip once but had as much time as they required after the clip finished to complete their ratings. Participants first rated the pain of the child in the given video clip and then rated the child’s fear. A research assistant was present in the room while participants completed this task to answer any questions.

### Video stimuli

The children in the video clips represent a sample of 20 boys (*n* = 10) and girls (*n* = 10) between the ages of 5 and 10 years who were videotaped during venipuncture as part of a previous study^20^ and whose parent provided consent to have their videos used for future research purposes. Children in the video were not related to the participants who were providing the pain and fear ratings in the current study. The children in the videos were chosen to present varying levels of pain, as indicated by their self-report rating of pain using the Faces Pain Scale–Revised^21,22^ following their procedure (where none = face 1, *n = *5; low = faces 2–3, *n = *5; medium = faces 4–5, *n = *5; high = face 6, *n* = 5). Independent samples *t*-tests revealed no significant differences in self-reported pain, *t*(18) = 0.33, *P* > 0.05, or fear ratings, *t*(18) = −0.17, *P* > 0.05, according to child sex.

### Measures

#### Anxiety

Participants’ trait anxiety was measured using the Trait subscale of the State-Trait Anxiety Inventory for Adults, which has demonstrated reliability and validity.^23^ The Trait subscale measures an individual’s inclination toward experiencing anxiety and is considered to reflect a persistent individual characteristic.^23^ Participants were asked to indicate the degree to which a series of 20 statements reflect how they typically feel on a scale from *almost never* (1) to *almost always* (4). Total scores for the Trait subscale were calculated (see Table 1 for descriptive data).

**Table 1.** Descriptive summary of questionnaire measures.

Questionnaire	Mean	SD	Potential range	Observed range
Trait Anxiety^a^	36.11	8.77	20–80	20–68
Parental Pain Catastrophizing^b^	20.84	8.63	0–52	2–45
Dispositional Empathy^c^				
Perspective Taking	18.91	4.52	0–28	6–28
Fantasy	14.17	5.60	0–28	1–27
Empathic Concern	20.89	4.23	0–28	8–28
Personal Distress	8.04	4.94	0–28	0–27

^a^Scores for trait anxiety were measured using the State-Trait Anxiety Inventory for Adults.

^b^Scores for parental pain catastrophizing were measured using the Pain Catastrophizing Scale for Parents.

^c^Scores for dispositional empathy were measured using the Interpersonal Reactivity Index.

#### Parental pain catastrophizing

The trait version of the Pain Catastrophizing Scale for Parents is a measure of parents’ tendencies to catastrophize about their children’s pain, with evidence for reliability and validity in parents of children and adolescents.^24^ Participants were asked to rate the degree to which they endorsed 13 statements reflecting catastrophic thoughts and feelings a parent might have in response to their child’s pain on a scale from *not at all* (0) to *extremely* (4). Participants’ total scores on this measure were calculated (see Table 1 for descriptive data). Participants completed this questionnaire with respect to their own children. Although participants were unrelated to the children whose pain and fear they were assessing, this measure was completed to provide an estimate of a participant’s tendency to catastrophize about a child’s pain.

#### Empathy

Dispositional empathy was assessed using the Interpersonal Reactivity Index,^25,26^ which has demonstrated reliability^26^ and validity.^25^ This measure is composed of 28 items that are divided equally across four subscales: (1) The Perspective Taking subscale examines an individual’s ability to take on the perspective of another; (2) the Fantasy subscale assesses an individual’s inclination to become absorbed into fictional characters or scenarios; (3) the Empathic Concern subscale examines an individual’s disposition toward experiencing empathic feelings (e.g., concern) toward others; and (4) the Personal Distress subscale measures an individual’s tendency to respond with feelings of self-oriented upset in response to emergency and intense emotional situations.^25,26^ Participants are asked to indicate how well each statement describes them on a scale from *does not describe me well* (0) to *describes me very well* (4). Scores were calculated for each subscale (see Table 1 for descriptive data).

#### Child pain intensity

Participants were asked to rate the pain of each child in the video using the Faces Pain Scale–Revised (FPS-R), which is widely used and has demonstrated evidence for being a valid measure of young children’s pain.^21,22^ This instrument is composed of six faces illustrating *no pain* to *very much pain*, with scores ranging from 0 to 10. Participants were instructed to circle the face that showed how much pain they thought the child in the video clip felt. The children in the video clips had also previously provided self-report ratings of pain using the FPS-R (see Table 2 for a summary of child pain ratings). Observers have similarly been asked to use child pain measures to provide ratings of children’s pain in other judgment studies.^1,3^

**Table 2.** Summary of pain and fear ratings self-reported by children in the video clips by child sex.

Participant sex	Pain level	FPS-R	CFS
Female	None	0	0
Female	None	0	0
Female	None	0	0
Female	Low	2	1
Female	Low	4	1
Female	Medium	6	2
Female	Medium	6	1
Female	Medium	8	2
Female	High	10	4
Female	High	10	1
Male	None	0	0
Male	None	0	0
Male	Low	2	0
Male	Low	2	0
Male	Low	4	1
Male	Medium	6	3
Male	Medium	8	1
Male	High	10	3
Male	High	10	3
Male	High	10	0

FPS-R = Faces Pain Scale–Revised; CFS = Children’s Fear Scale.

#### Child fear

Participants rated the fear of the children in the video using the Children’s Fear Scale (CFS).^7^ This measure depicts five faces ranging from *not scared at all* to *the most scared possible* and has demonstrated reliability and validity as a tool for assessing children’s procedure-related fear.^7^ Participants were asked to circle the face that showed how scared they thought the child in the video clip was, with scores ranging from 0 to 4. The children in the video clips had also previously provided self-report ratings of fear using the CFS (see Table 2 for a summary of child fear ratings).

#### Pain judgment accuracy

An overall percentage of accurate agreements (i.e., a rating of the same face on the FPS-R as the child had circled) with child pain intensity ratings for boys (out of ten ratings) and girls (out of ten ratings) were calculated for each participant. Overall percentage scores for underestimation and overestimation of child pain ratings were also calculated for each participant. These percentage scores took into account that it would not be possible for a participant to underestimate a child who rated his or her pain as the first face on the FPS-R (*n = *5) or, similarly, to overestimate a child who rated his or her pain as the last face on the FPS-R (*n = *5). Specifically, underestimation was defined as a rating lower than the child’s self-reported pain and could occur for eight out of ten boys and seven out of ten girls. Similarly, overestimation was defined as any rating higher than the child’s self-reported pain, which could occur for seven out of ten boys and eight out of ten girls. The camera angle and quality of the video clips were not conducive to facial coding; therefore, participant judgment accuracy was based solely on the child’s self-report rating (which is regarded as a primary method of child pain assessment^8^).

#### Fear judgment accuracy

Using the same approach as that described for pain judgment accuracy, overall percentage scores were calculated for accurate agreements with child fear ratings (i.e., rating the same face on the CFS as the child had self-reported) for boys (out of ten ratings) and girls (out of ten ratings) for each participant. Overall percentage scores for underestimation (i.e., a rating lower than the child’s self-reported fear) and overestimation (i.e., a rating higher than the child’s self-reported fear) were also calculated for boys and girls for each participant. Of note, the children included in the video stimuli were not stratified for level of self-reported fear and many of the children indicated low fear ratings. Thus, participants had more opportunity to overestimate boys’ (calculated out of ten children’s ratings) and girls’ (calculated out of nine children’s ratings) fear than to underestimate boys’ (calculated out of five children’s ratings) and girls’ (calculated out of seven children’s ratings) fear.

### Analytic plan

The impact of participant and child sex on participant judgment accuracy of children’s pain and fear was analyzed using 2 (between: participant sex) × 2 (within: child sex) mixed analyses of variance. To investigate whether the variables of interest predicted participant accuracy differentially between women and men, a series of multiple regression analyses was conducted separately for women and men with trait anxiety, parental pain catastrophizing, and the four empathy constructs (perspective taking, fantasy, empathic concern, and personal distress) entered all at once as predictor variables and the percentage of accurate agreement, overestimation, and underestimation of child pain and fear entered separately as the respective dependent variables. In cases where neither regression model for women and men was significant, the multiple regression analyses were repeated in the same manner described above separately for boys and girls, with women and men combined (i.e., the respective dependent variables were percentage of accurate agreements, overestimation, and underestimation of boys’ and girls’ pain and fear, each examined separately). In cases where neither model was significant (i.e., for boys or girls), the regression analyses were conducted with the data from boys and girls combined.

## Results

### Adult judgment of children’s pain

Examination of the mean percentage agreement scores suggests that participants in the current sample had generally low levels of agreement with children’s pain ratings (see Table 3) and often underestimated children’s pain (see Table 4). A significant main effect of child sex was discovered, F(1,158) = 41.70, *P* < 0.001, partial η^2^ = 0.21, with participants accurately judging boys’ pain significantly more often than girls’ pain. No main effects of participant sex or interactions emerged. To explore whether child sex differences existed in the types of errors participants made when judging children’s pain, paired samples *t*-tests were conducted with data from women and men combined. These analyses revealed that participants both underestimated, *t*(159) = −3.69, *P* < 0.001, and overestimated, *t*(159) = −8.65, *P* < 0.001, girls’ pain significantly more frequently than boys’ pain.

**Table 3.** Mean percentage (and standard deviations) of accurate participants’ judgments of children’s pain and fear by adult and child sex.

	Pain			Fear		
Child sex	Women	Men	Combined	Women	Men	Combined
Boy	34.63 (15.65)	35.51 (14.38)	35.06 (15.00)	42.19 (14.66)	42.44 (15.13)	42.31 (14.85)
Girl	25.73 (13.15)	25.26 (13.55)	25.50 (13.31)	36.71 (13.34)	34.87 (13.17)	35.81 (13.24)

**Table 4.** Mean percentage (and standard deviations) of participants’ underestimation and overestimation of children’s pain and fear by child sex.

	Pain		Fear	
Child sex	Underestimation	Overestimation	Underestimation	Overestimation
Boy	59.06 (22.69)	25.27 (18.77)	29.25 (16.92)	43.06 (16.75)
Girl	64.37 (19.69)	36.80 (17.13)	27.41 (15.61)	50.00 (16.39)

### Adult judgment of children’s fear

Examination of the mean percentage agreement scores for participant ratings of children’s fear indicated low levels of agreement (see Table 3), with participants often overestimating children’s fear (see Table 4). A significant main effect of child sex was discovered, F(1,158) = 19.75, *P* < 0.001, partial η^2^ = 0.11, with participants accurately judging boys’ fear significantly more often than that of girls’. No main effects of participant sex or interactions were found. To explore whether the types of errors participants made when judging children’s fear differed by child sex, paired sample *t*-tests were conducted with data from women and men combined. These analyses revealed that participants overestimated girls’ fear significantly more frequently than boys’ fear, *t*(159) = −5.41, *P* < 0.001. However, there was no significant difference between participants’ underestimation of boys’ and girls’ fear, *t*(159) = 1.21, *P* = 0.23.

### Predictors of adult judgment of children’s pain

Neither of the models conducted separately for men and women was significant for accuracy (men: adjusted *R*^2^ = 0.024; women: adjusted *R*^2^ = −0.003), overestimation (men: adjusted *R*^2^ = 0.084; women: adjusted *R*^2^ = 0.011), or underestimation (men: adjusted *R*^2^ = 0.054; women: adjusted *R*^2^ = 0.033). Therefore, the regression analyses were repeated separately for boys and girls. The model was not significant for accuracy in judging boys’ (adjusted *R*^2^ = −0.017) or girls’ (adjusted *R*^2^ = 0.013) pain and was also not significant when conducted with participant and child sex combined (adjusted *R*^2^ = 0.017). The model was again not significant for overestimation of boys’ (adjusted *R*^2^ = 0.025) or girls’ (adjusted *R*^2^ = 0.002) pain and was also not significant when conducted with participant and child sex combined (adjusted *R*^2^ = 0.022). Although the model was not significant for underestimation of boys’ pain (adjusted *R*^2^ = 0.019), the model was significant for underestimation of girls’ pain. The variables together predicted 7.6% of the variance in percentage of participant underestimation of girls’ pain, adjusted *R*^2^ = 0.076, F(6,153) = 3.17, *P* < 0.01. Parental pain catastrophizing was the only significant predictor variable (standardized β = −0.35, *P* < 0.001) and was significantly negatively correlated with participant underestimation of girls’ pain (*r* = −0.29, *P* < 0.001).

### Predictors of adult judgment of children’s fear

Each model was not significant when conducted separately for women (accuracy: adjusted *R*^2^ = −0.041; overestimation: adjusted *R*^2^ = −0.056; underestimation: adjusted *R*^2^ = 0.001) or men (accuracy: adjusted *R*^2^ = −0.032; overestimation: adjusted *R*^2^ = −0.009; underestimation: adjusted *R*^2^ = −0.05), for boys (accuracy: adjusted *R*^2^ = 0.005; overestimation: adjusted *R*^2^ = 0.01; underestimation: adjusted *R*^2^ = −0.02) or girls (accuracy: adjusted *R*^2^ = −0.025; overestimation: adjusted *R*^2^ = −0.002; underestimation: adjusted *R*^2^ = −0.021), or with the data combined (accuracy: adjusted *R*^2^ = −0.008; overestimation: adjusted *R*^2^ = −0.001; underestimation: adjusted *R*^2^ = −0.015).

### Post hoc analyses

Additional post hoc analyses were conducted to further examine the relationship between adult judgements of children’s pain and fear. The correlation between adult pain and fear ratings was examined within each child, and the mean correlation across the 20 children was *r* = 0.44, suggesting a medium to large effect.^27^ Correlational analyses were also conducted between adult overestimation of girls’ fear and adult over- and underestimation of girls’ pain. Adult overestimation of girls’ fear was significantly positively related to overestimation of girls’ pain (*r* = 0.53, *P* < 0.01) and significantly negatively related to underestimation of girls’ pain (*r* = −0.44, *P* < 0.01).

## Discussion

This study built on prior work examining caregiver–child agreement in pain assessment through exploring factors that may explain caregiver misestimation. This study also offered a novel examination of caregiver judgment accuracy of children’s pain-related fear, which is an important aspect of children’s pain experiences and treatment. Specifically, this study explored the impact of adult and child sex on judgments of children’s pain and fear during venipuncture and examined whether traits of parental pain catastrophizing, empathy, and anxiety were predictors of participants’ judgment accuracy using a standardized methodological approach. In line with previous research,^1–6^ this study found that participants had generally low levels of agreement with child reports of pain during venipuncture, most often underestimating both boys’ and girls’ pain. Further, similar to McMurtry and colleagues,^7^ participants also had low agreement with children’s fear ratings. However, participants tended to overestimate children’s procedural fear.

Contrary to Moon and colleagues’ findings,^3^ there were no differences between women’s and men’s accuracy in judging children’s pain or fear, nor were there any interactions between adult and child sex. However, child sex did influence judgments, with ratings of boys’ pain being more accurate than ratings of girls’ pain. Further, participants were more likely to both overestimate and underestimate girls’ pain relative to boys’ pain, suggesting that adults are misestimating girls’ pain in both directions. Similarly, participants were more accurate in judging boys’ fear relative to that of girls. However, participants seemed to more systematically overestimate girls’ fear relative to that of boys. These findings establish child sex as a relevant factor in understanding parental misestimation of both child pain and fear.

It has been suggested that adults may rate boys’ pain higher than girls’ pain because gender stereotypes typically indicate that girls are expected to express their pain to a greater degree than boys, thus causing adults to interpret boys’ pain behaviors as signifying more pain than analogous behaviors by a girl.^3,12^ Concurrent with this idea, it is possible that adults assign more importance to boys’ expressions of pain or fear because it is less expected, resulting in more accurate ratings. The fact that participants both over- and underestimated girls’ pain more often than boys suggest that adults may experience uncertainty regarding how to interpret girls’ pain behaviors, leading to more random judgments. It is also possible that girls’ pain displays may be more contradictory (e.g., verbally expressing pain while nonverbally indicating little pain) or fluctuate in intensity more frequently than boys, making it more challenging to assess. Further research would be needed to examine this. Additionally, adults’ overestimation of girls’ fear seems to be in part related to their misestimation of girls’ pain. Adults who were more likely to overestimate girls’ fear were also more likely to overestimate girls’ pain. If an adult believes that a child’s displays of fear are also indicative of his or her pain level, overestimation of fear could lead to overestimation of pain, which could result in mismanagement of both. This finding may also suggest that some parents generally tend to overrate girls’ distress, with regard to both pain and fear.

It was hypothesized that participant traits of parental pain catastrophizing, anxiety, and empathy would significantly predict their judgments of children’s pain and fear. Only parental pain catastrophizing emerged as a significant predictor of participants’ underestimation of girls’ pain. Concurrent with previous findings,^4^ a negative relationship was found between participants’ parental pain catastrophizing and underestimation, suggesting that participants who reported higher levels of catastrophizing about their own child’s pain were less likely to underestimate other female children’s pain. It is possible that parental pain catastrophizing may serve as a buffer against a tendency to underestimate girls’ pain, resulting in more accurate ratings. Given that this finding was discovered only for girls, future research should explore predictors of underestimation of boys’ pain.

The results of the present study have important implications for clinical pain and fear assessment. Due to the heterogeneity of pediatric patients and caregivers presenting in clinical contexts, understanding individual difference factors (e.g., sex, anxiety) that may impact adult judgments of child pain and fear is necessary to improve pediatric pain assessment and management. Given that even low levels of pain or fear may be clinically meaningful to any one child,^28,29^ inaccurate assessment by a caregiver could have significant implications for a child receiving the pain or fear management that he or she needs. The finding that participants generally had low levels of agreement with children’s pain and fear ratings suggests that caregiver report should not be interpreted as a direct representation of a child’s perspective of his or her pain experience. However, caregivers are an important component of young children’s health care. Therefore, health care professionals should take children’s self-report into consideration as well as parent report when possible in order to obtain the best understanding of a child’s pain-related experience, consistent with a recommended broad approach to child pain assessment.^8^ This may be particularly important for girls, given adults’ tendencies to be less accurate when judging girls’ pain and fear relative to boys’ pain and fear. As suggested by Cohen and colleagues,^12^ differences in how adults rate boys’ and girls’ pain could lead to differences in pain treatment. The findings of the current study suggest that girls may be particularly susceptible to inappropriate management of their pain and fear. There is evidence in the adult literature to suggest that women obtain poorer pain management relative to men (Hoffman and Tarzian^30^ provide a review). Systematic misestimation of girls’ pain and fear, even if experienced at low levels, may possibly contribute to poorer pain management over time. Examining factors related to inaccuracy in the assessment of girls’ pain and fear may be a starting point to addressing sex differences in pain treatment across the life span.

The judgment task used in the present study had several strengths, including the use of the same rating scales for adults and children^6^ and the portrayal of children actually undergoing a common medical procedure. The standardized methodological approach, including videos that systematically manipulated child sex and self-reported pain level, allowed for the examination of factors that may impact adult judgments of child pain in a more controlled manner than could have been achieved in a clinical setting. However, there are also several notable limitations. Several factors may have impacted the generalizability of the results. The adults who participated in the study were unrelated to the children in the videos and therefore were not assessing their own children, and it is possible that this may have impacted their pain and fear ratings, limiting the generalizability of the findings. For example, parents viewing their own child would be familiar with their child’s typical pain and fear responses and would likely have an emotional connection with their child, which could potentially increase or decrease the accuracy of their assessments. Additionally, parents’ pain and fear assessments in real-world settings would be informed by more information than that provided in a 10-s period such as that portrayed in the current video clips (e.g., the pain context, child behaviors prior to the painful experience). The findings are also limited to the age range of the children in the video clips and may not generalize to older or younger children. The child’s parent and health care professionals were often visible in the videos, and the behaviors of these individuals may have influenced participants’ child pain ratings; however, this influence may be similar to that in clinical settings. The limitations associated with the video portrayals may be particularly salient given that parents only viewed a sample of 20 video clips.

Limitations also exist with regard to the study methodology. A strict definition of accuracy as exact agreement was used in the current study. It is possible that the influence of caregiver–child agreement on clinical pain and fear management is more complex than that captured by the current definition. Children’s pain and fear were measured using only self-report; thus, the children’s behaviors or facial expressions and their potential impact on participants’ judgments were not taken into consideration in analyses. Further, it is possible that questionnaires alone are not the most salient way of assessing the variables that might influence adults’ child pain and fear judgments. Participants also completed the catastrophizing measure with respect to their own child. The participants completed the pain and fear rating task reported on in this study after completing an experimental pain task with their own children. It is possible that their children’s and their own responses during this pain task (e.g., coping, anxiety), as well as their own children’s sex, may have impacted participants’ pain and fear ratings when watching the video clips. Significant differences were found between women and men in the current sample with regard to age and marital status, which may have influenced the findings regarding participant sex. Lastly, a large number of regression analyses were conducted, which may have increased the probability of a spurious finding.

Given the importance of accurate pain assessment in children’s health care, future research should continue to provide insight on the processes involved in pain and fear assessment. Anxiety and dispositional empathy were not predictors of participants’ judgment accuracy in the current study, and parental pain catastrophizing only accounted for a small amount of variance in underestimation of girls’ pain. Future research could explore these variables in a clinical context, as well as other variables that might predict caregivers’ estimations of children’s pain and fear, such as parents’ emotion regulation abilities^31^ or their own responses to pain (e.g., parent pain tolerance, their own pain catastrophizing^16^). Further, trait measures of anxiety, parental pain catastrophizing, and empathy were examined in the current study. However, it is possible that adults’ state or “in-the-moment” experiences of these constructs may influence assessment of children’s pain and fear and should be explored in future research. Child pain behaviors, such as their facial expressions, may also be a relevant variable to explore.^4^ Comparing boys’ and girls’ behaviors during pain may provide insight into why caregivers tend to misestimate girls’ pain and fear. Exploring the influence of child sex on pain and fear assessment within the context of other factors known to influence pain judgments, such as chronic versus acute pain^10^ and child age,^11^ would also be valuable. Further, examining the clinical relevance of the degree of discrepancy in caregiver–child pain and fear ratings may be valuable. Other methodologies could be used to further understand child pain assessment. For example, an electroencephalographic study found support for women demonstrating greater empathy when viewing someone experiencing pain than men.^32^ Eye-tracking equipment could provide information on what caregivers attend to while watching a child in pain.^33^ Further, a “think-aloud” procedure could be used while caregivers are watching a child in pain; the verbal information could be coded and compared between caregivers who accurately assess and those who misestimate their child’s pain, and caregiver and child sex differences could be explored. Future studies could also examine parent and child sex differences in mothers’ and fathers’ pain and fear assessments for their own children and compare siblings.

In conclusion, the current study provides a novel contribution to our understanding of adult judgments during painful medical procedures. The study examined factors that may contribute to adult misestimation, including adult and child sex and adult traits of parental pain catastrophizing, empathy, and anxiety. Further, this study examined how these factors may influence fear ratings in addition to pain, which represents an underresearched area in the field. The results of this study suggest that child sex has an influence on adults’ assessments of children’s pain and fear, with both women and men being more accurate at assessing boys’ pain and fear compared to girls’ pain and fear. From a theoretical perspective, these findings can inform interpersonal and social/communication theories regarding pediatric pain assessment. Clinically, these findings indicate that girls may be vulnerable to inaccurate assessment of their procedural pain and fear.
